# Attempts to Detect Lipid Metabolites from a Single Cell Using Proton-Transfer-Reaction Mass Spectrometry Coupled with Micro-Scale Supercritical Fluid Extraction: A Preliminary Study

**DOI:** 10.5702/massspectrometry.A0112

**Published:** 2022-12-29

**Authors:** Toshinobu Hondo, Chihiro Ota, Kohta Nakatani, Yumi Miyake, Hiroshi Furutani, Takeshi Bamba, Michisato Toyoda

**Affiliations:** 1 MS-Cheminformatics LLC, 2–13–21 Sasao-nishi, Toin, Inabe, Mie 511–0231, Japan; 2 Forefront Research Center, Graduate School of Science, Osaka University, 1–1 Machikaneyama, Toyonaka, Osaka 560–0043, Japan; 3 Graduate School of Science and Engineering, Kansai University, 3–3–35 Yamate-cho, Suita, Osaka 564–8680, Japan; 4 Division of Metabolomics, Medical Institute of Bioregulation, Kyushu University, 3–1–1 Maidashi, Higashi-ku, Fukuoka 812–8582, Japan; 5 Center for Scientific Instrument Renovation and Manufacturing Support, Osaka University, 1–2 Machikaneyama, Toyonaka, Osaka 560–0043, Japan

**Keywords:** supercritical fluid extraction, supercritical fluid chromatography, single-cell lipid metabolite analysis, proton-transfer-reaction mass spectrometry, water ion chemical ionization

## Abstract

Proton-transfer-reaction (PTR) mass spectrometry (MS), a widely used method for detecting trace-levels of volatile organic compounds in gaseous samples, can also be used for the analysis of small non-volatile molecules by using supercritical fluid as a transporter for the molecules. Supercritical fluid extraction (SFE) is a method that permits lipophilic compounds to be rapidly and selectively extracted from complex matrices. The combination of the high sensitivity of PTR MS with the SFE is a potentially novel method for analyzing small molecules in a single cell, particularly for the analysis of lipophilic compounds. We preliminarily evaluated this method for analyzing the components of a single HeLa cell that is fixed on a stainless steel frit and is then directly introduces the SFE extracts into the PTR MS. A total of 200/91 ions were observed in positive/negative ion mode time-of-flight mass spectra, and the masses of 11/10 ions could be matched to chemical formulae obtained from the LipidMaps lipids structure database. Using various authentic lipophilic samples, the method could be used to detect free fatty acids in the sub-femtomole to femtomole order in the negative ion mode, the femtomole to sub-picomole order for fat-soluble vitamins, and the picomole order for poly aromatic hydrocarbons in both the positive and negative ion mode.

## 1. INTRODUCTION

Immortalized cells such as HeLa, HepG2, and Huh-7 cells have been widely used by researchers in the molecular biological sciences^1–8)^ because they are essentially identical cells that never change. The use of such cells has contributed to the study of tumor tissue formation at the molecular level. Because the enzymes and their substrates that are involved in the regulation of metabolic pathways for growing or suppressing tumors are present at trace levels, a collection of identical cells has long been essential for studies in this area. Pathological tumor tissue, however, is comprised of a heterogeneous collection of cells. Because of this, understanding the metabolism in a single cell and identifying the difference between cells and their population and cell-to-cell signaling systems is a crucial issue. Various lipid-soluble compounds, especially free fatty acids (FFAs), play significant roles in mammalian bodies and are involved in many diseases or disorders, such as cancers, fever, pain, and bipolar disorders.^9–14)^ However, their measurement in a single cell is not always easy due to the small amounts of these substances, the presence of isomers, and the complex sample preparation procedures.

Microscale supercritical fluid extraction is a potential candidate for preparing samples for the analysis of lipophilic molecules in a formed/structured entity such as a cell. The method permits the selective extraction of lipophilic compounds from a complex sample matrix by controlling the temperature and pressure with an entrainer, when necessary. Ivanov *et al.*^15)^ reported that SFE is a well-suited and recommended method for preparing samples for fatty acid analysis. Proton-transfer-reaction (PTR) ionization-mass spectrometry (MS) is a method that is well-suited for detecting molecules in a supercritical fluid without the need for sophisticated instrumentation.^16)^ In this method, a sample is applied on a stainless steel frit that is directly introduced into a PTR mass spectrometer by supercritical carbon dioxide (scCO_2_) without further sample preparation. The PTR MS that we designed, essentially involves water positive/negative ion chemical ionization and has advanced detection sensitivity for both positive and negative ions, which are produced simultaneously.^17)^ It is tremendously advantageous for detecting FFAs with a high sensitivity and appears to show a better sensitivity in the negative ion mode.

## 2. EXPERIMENTAL

### 2.1. Apparatus

**Figure 1:**
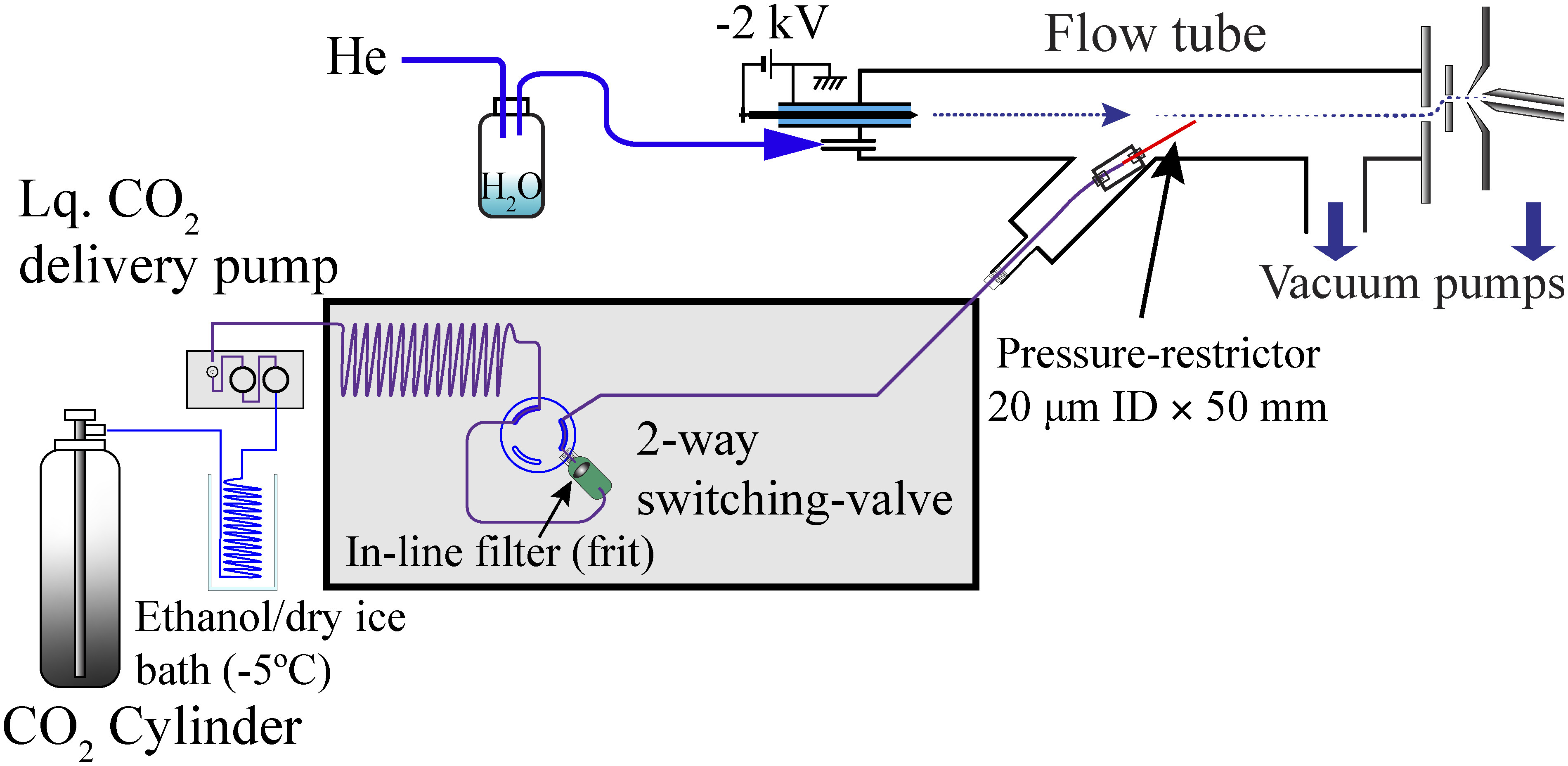
Fig. 1. Block diagram of SFE-PTR MS.

The apparatus used in this study was the same as previously reported^16)^ as the “Direct injection” (DI) method. The set up consists of a JMS-T100 LP (AccuTOF) time-of-flight (TOF) mass spectrometer (JEOL, Tokyo, Japan) with an altered data acquisition system using the Acqiris Model U5303A with QtPlatz (https://github.com/qtplatz/qtplatz) open source software. The data acquisition system simultaneously enables “peak detection” (PKD) and waveform averaging (AVG).^18,19)^ Figure 1 illustrates a block diagram of the apparatus, which consists of an in-house constructed PTR flow tube, an SFE interface, and an AccuTOF interface. Liquid CO_2_ from a cylinder (Iwatani Industrial Gases Corp., Osaka, Japan) was precooled to −5°C using an ethanol/dry ice bath and then pressurized up to 25 MPa at a rate of 500 μL·min^−1^ by an LC Packings UltiMate Micropump (Thermo Scientific, MA, US). The pressurized carbon dioxide from the pump was connected to the Agilent 1100 Series Thermostatted Column Compartment (Agilent, CA, US) and a Rheodyne 7000 switching valve was connected through a microscale extraction vessel.

An inactivated fused silica capillary (GL Science, Tokyo, Japan) approximately 50 mm in length with a 20 μm inner-diameter (ID) was used as a scCO_2_ pressure restrictor, which holds up to 30 MPa at a liquid CO_2_ flow rate set at 0.5 mL·min^−1^ at the high-pressure end with the other end open to the PTR flow tube, which is at approximately 100 Pa.

General grade helium (Iwatani Industrial Gases Corp., Osaka, Japan) was connected to a flow tube after passing through a 250 mL volume of the solvent-reservoir bottle containing 10 mL of ultrapure water (Millipore, MA, US). The helium flow rate was set to 70 mL·min^−1^, which was controlled using a mass flow controller (MFC) (8500MC-0-1-2, KOFLOC Corp., Kyoto, Japan). The pressure of the flow tube was maintained in a range of 90 to 105 Pa by an AccuTOF vacuum system. The model PS350 high voltage power supply (Stanford Research Systems, Inc., Sunnyvale, CA, US) was used as a corona discharge power supply. The voltage for the discharge electrode was set to −2.0 kV, which resulted in 80 to 100 μA of current.

The pump head for CO_2_ delivery was cooled to approximately 8°C by the Peltier module (Hebei, TES1-12705, Hebei, China) prepared in-house.

An ACQUITY column in-line filter (Waters, MA, US) connected to a 2-way switching valve was used as the SFE vessel.

### 2.2. Chemicals

Hexamethoxyphosphazine (P321), Hexakis (2,2-Difluoroethoxy) phosphazene (P621), tetracosenoic acid, caffeine, pyrene, reserpine, oleic acid, vitamin K_1_, and γ-oryzanol were purchased from FUJIFILM Wako Pure Chemicals Corporation, Osaka, Japan. Methanol, acetonitrile (LC/MS grade), and reagent-grade acetone (FUJIFILM Wako Pure Chemicals Corporation, Osaka, Japan) were used as a solvent for the above chemicals. Water was obtained from a Milli-Q Purification System (Merck, Germany). Cylinders of general grade helium and carbon dioxide (siphon type) (Iwatani Industrial Gases Corp., Osaka, Japan) were used in this study.

### 2.3. Mass calibration

Mass calibration was performed using sodium trifluoroacetate, an electrospray ionization (ESI) source for *m*/*z* between 159 and 703 using third-order polynomials. The PTR ion source was then attached by altering the ESI source.

### 2.4. HeLa cell sample preparation

HeLa cells were prepared as described in a previous report.^20)^ The cells (American Type Culture Collection) were cultured in 10 cm diameter dishes containing 10 mL of Dulbecco’s modified Eagle’s medium (DMEM, Thermo Fisher Scientific, Inc., Waltham, MA, USA) supplemented with 10% (v/v) fetal bovine serum (FBS, Thermo Fisher Scientific, Inc.) and 1% (v/v) Penicillin–Streptomycin Solution (Thermo Fisher Scientific, Inc.) as antibiotics. Cultivation dishes were incubated in a water-jacketed CO_2_ incubator (WCI-165; ASTEC Co., Fukuoka, Japan) under an atmosphere of 5% CO_2_ at 37°C. When the cells reached 80% confluence, the medium in each dish was changed 1 h before cell sampling. The cultivated HeLa cells in 10 cm dishes were harvested at an 80% confluency by treatment with a Trypsin–EDTA solution for 3 min at 37°C. After counting cell numbers using a cell counter (Moxi Z; ASONE Co., Osaka, Japan), the Trypsin–EDTA-treated HeLa cells were collected in a 15-mL tube by centrifugation with a swing type rotor at 250×g for 1 min at 20°C. The resulting cell pellets were washed four times with 10 mL PBS. The PBS aliquot was then added to the cell pellets, and 1×10^−4^ cells·mL^−1^ cell suspension was prepared. Single HeLa cells were aspirated from the suspension in the fused-silica capillary (100 μm i.d., 360 μm o.d., 75 mm length). After microscopic confirmation of a single HeLa cell, the isolated cell was ejected on the frit and then dried.

Another batch of HeLa cell pellets was prepared with the same protocol described above. After adding 1 mL of methanol to the HeLa cell pellets, the samples were vigorously mixed by vortexing for 1 min, followed by sonication for 5 min. After centrifugation for 5 min at 16,000×g and 4°C, 700 μL of the supernatant was transferred to a 2 mL Eppendorf tube. A portion of the supernatant was appropriately diluted with methanol to make 100 cells·μL^−1^, and this was used as a methanol-extract of a cell.

### 2.5. SFE-PTR MS analysis procedure

A portion of the lock-mass reference sample was applied to the frit that holds a single HeLa cell, as described above. After the acetonitrile had evaporated, the frit was set to the Acquity In-Line filter and the SFE was started by changing the 2-way valve position. SFE-PTR MS spectra were acquired using simultaneous PKD and AVG waveforms for intervals of 0.15 s, and were stored as a function of time. An extracted ion profile of an ion should show a bell-shaped curve (broad peak), which can be found by the peak detection algorithm. In contrast, the ion peaks derived from reagent ions and the intensity of the common instrumental background ions are maintained at the beginning and then decrease when the sample-derived ions begin to arrive. By using a chromatographic peak detection algorithm, the ions that show peak-like shapes were highlighted in magenta,^21)^ indicating that the magenta color-coded ions appear to be derived from the SFE sample. The color-coded HeLa cell spectrum was then compared with the spectrum obtained when the HeLa cell was not on the frit, which contains the PBS buffer and the lock-mass reference sample. The ion peaks that were common to both the HeLa cell spectrum and no HeLa cell spectrum within ±5 mDa are highlighted in gray. All the magenta color-coded ion peaks were then compared with the structures listed in the LipidMaps database.

## 3. RESULTS AND DISCUSSION

### 3.1. Detection capability for authentic samples

**Figure 2:**
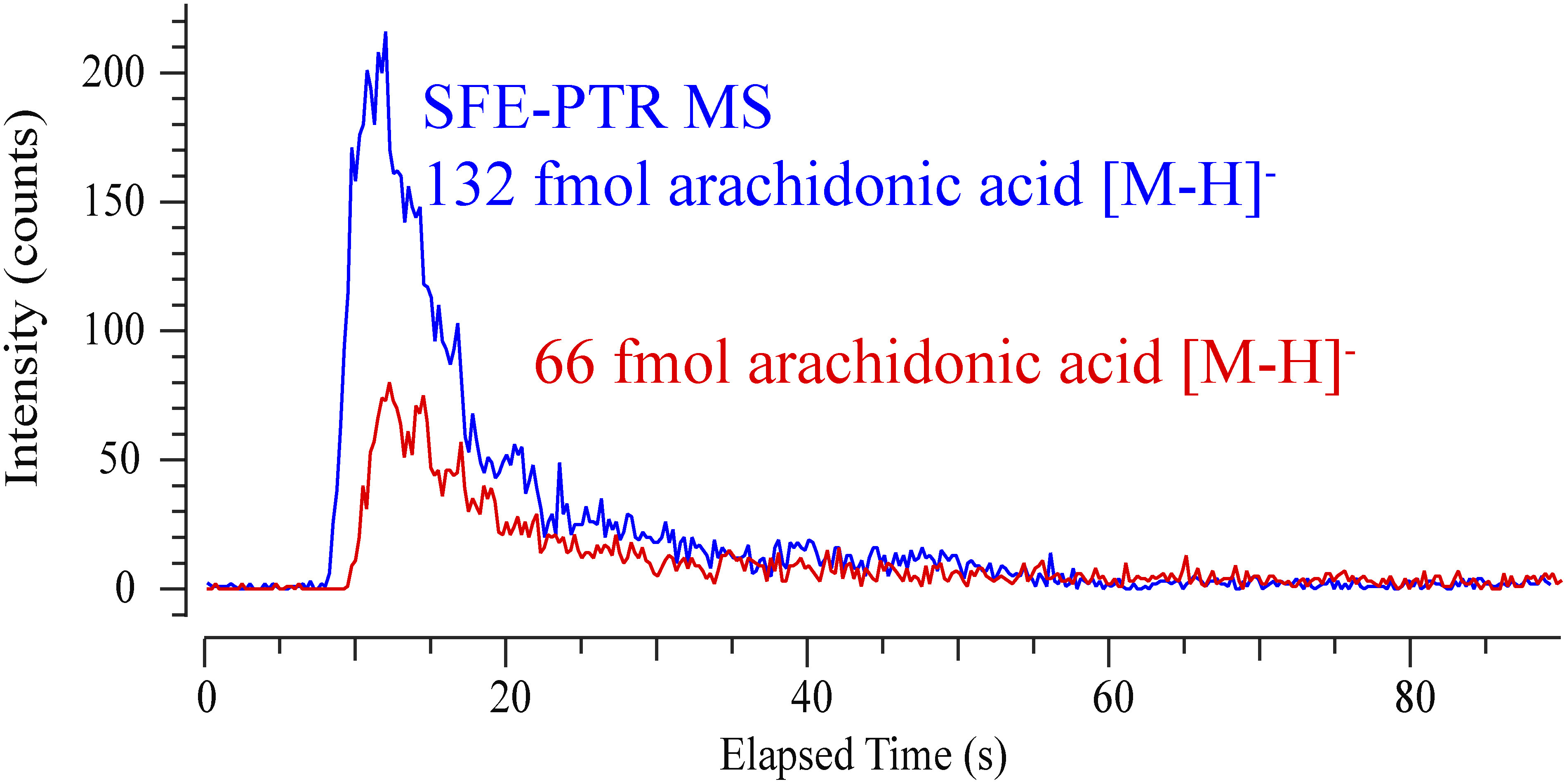
Fig. 2. Extracted ion profiles of SFE-PTR MS for 66 fmol (red) and 132 fmol (blue) of arachidonic acid in the negative ion mode.

We preliminarily tested the detection capability and sensitivity of the method using authentic samples. A list of compounds and the amounts needed to give an intensity (counts) of 100 are listed in Table 1. Each compound purchased from the supplier described in the Experimental section was dissolved in acetonitrile, and appropriately diluted samples were applied to the frit. After waiting a few minutes for the acetonitrile to evaporate, the frit was placed in the In-Line column filter, SFE was started so as to monitor the series of mass spectra as a function of time. Figure 2 illustrates the SFE profile for arachidonic acid as a typical cell component. The extracted ion profile of SFE for each ion was calculated, and the peak area was then obtained as the sum of ion counts within the half-height width region of the given extracted ion profile. The limit of quantitation was then calculated as the amount needed to produce 100 ion counts,^19)^ which were calculated from the slope of the sample amount *vs.* the number of ion counts for at least 2 or more different amounts of sample. The quantitative error for 100 counts is 10% (square root of 100), assuming that the counting result closely follows a Poisson distribution. The repeatability of the counts was approximately 50% of the relative standard deviation (RSD) due to the SFE pressure restrictor that occasionally became unstable. Nevertheless, the method showed an excellent detection sensitivity at the femtomole order for most of the medium to long-chain FFAs in the negative ion mode that were tested.

**Table 1:** Table 1. Detection capabilities for various authentic samples.

Compound	Mass	Positive ion mode		Negative ion mode	
Acetaminophen	151.063	4.9 fmol	[M+H]^+^	274 fmol	[M−H]^−^
Phenacetin	179.095	1.4 fmol	[M+H]^+^	203 fmol	[M−H]^−^
Caffeine	194.080	0.077 fmol	[M+H]^+^	—	
Pyrene	202.078	9.5 fmol	[M+H]^+^	—	
Oleic acid	282.256	—		0.048 fmol	[M−H]^−^
Stearic acid	284.272	—		10 fmol	[M−H]^−^
Linolic acid	280.240	—		0.20 fmol	[M−H]^−^
Arachidic acid	312.303	—		27 fmol	[M−H]^−^
Arachidonic acid	304.240	—		19 fmol	[M−H]^−^
Tetracosenoic acid	368.365	—		17 fmol	[M−H]^−^
α-Tocopherol	430.381	63 fmol	[M+H]^+^	4 fmol	[M−H]^−^
Vitamine K_1_	450.350	406 fmol	[M+H]^+^	41 fmol	[M]^−^
γ-Oryzanol	602.434	—		1.6 fmol	[M−H]^−^
Reserpine	608.273	8.3 fmol	[M+H]^+^	8000 fmol	[M−H]^−^
Ivermectin (B_1_*_a_*)	874.508	2.3 fmol	[M+NH_4_]^+^	—	

### 3.2. HeLa cell sample analysis

A total of 20 fmol of P321, P621 (positive ion mode), or 10 pmol tetracosenoic acid (negative ion mode) acetonitrile solution was added to the frit as the mass reference, which holds a single HeLa cell. After waiting a few minutes for the acetonitrile to evaporate, the frit was placed into the Acquity In-Line filter, and the “2-way switching valve” position was then switched to start the SFE. Three cells for each of the positive and negative ion modes were measured.

**Figure 3:**
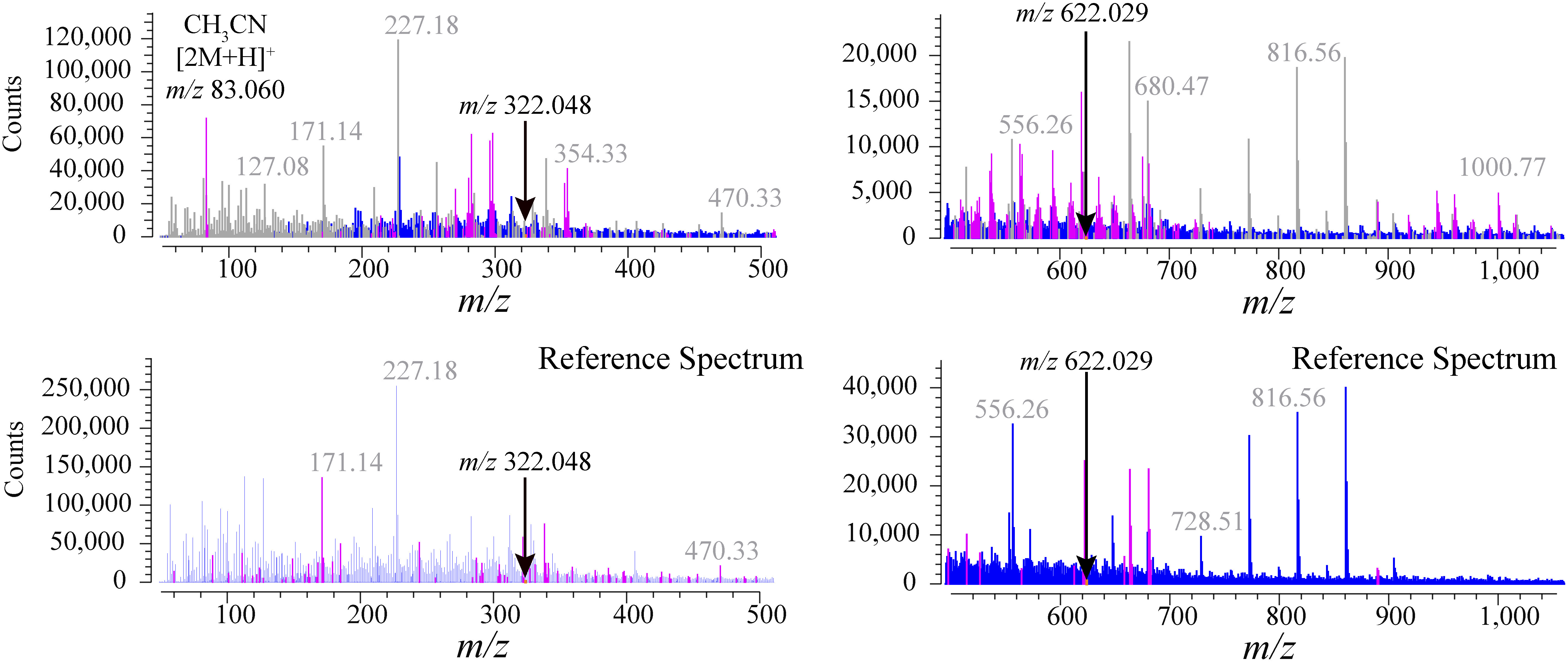
Fig. 3. Positive ion mode SFE-PTR mass spectrum of a cell on the frit (top). SFE-PTR spectrum of lock mass references sample (bottom). The spectrum data files are available in J-STAGE Data. https://doi.org/10.50893/data.massspectrometry.21706661

**Figure 4:**
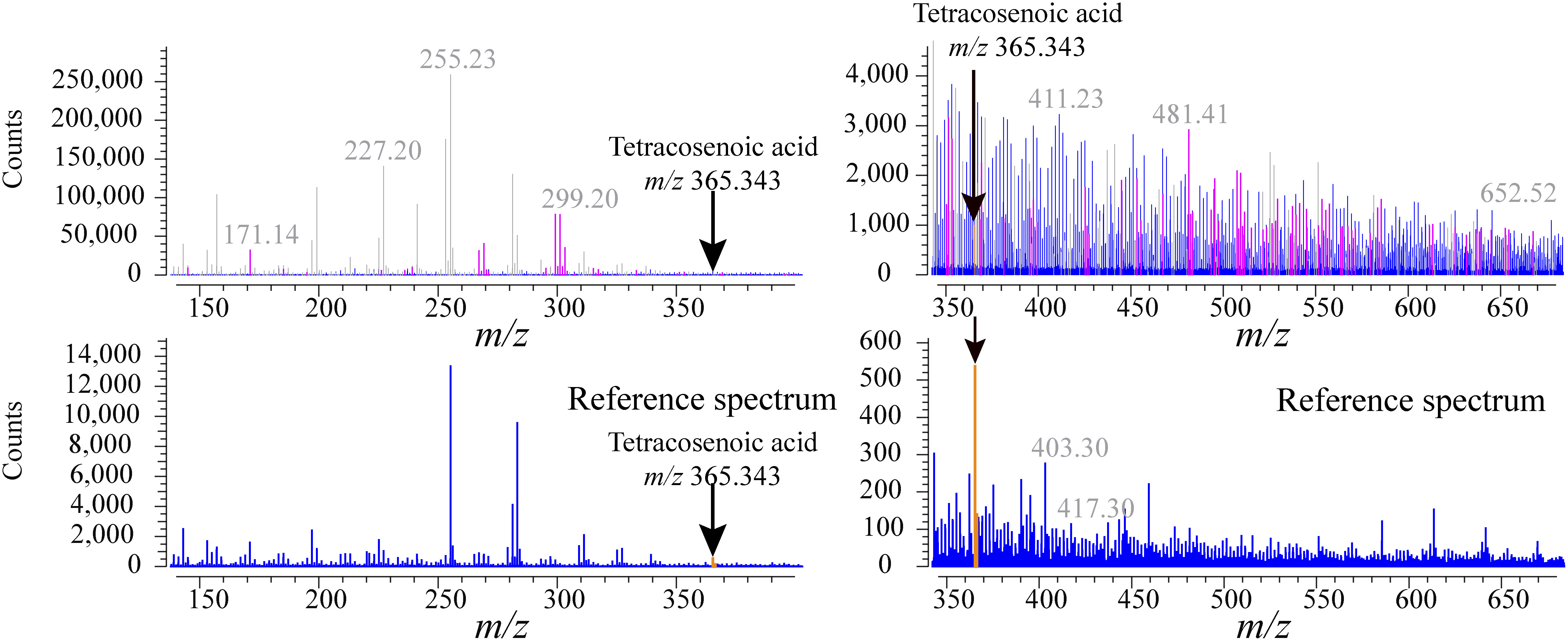
Fig. 4. Negative ion mode SFE-PTR mass spectrum of a cell on the frit (top). SFE-PTR spectrum of lock mass reference sample (bottom). The spectrum data file is available in J-STAGE Data. https://doi.org/10.50893/data.massspectrometry.21706664

Figures 3 and 4 are the co-added SFE-PTR mass spectra for a single HeLa cell SFE (top). The steps to obtain these spectra and the definition of the color code are described in Section 2.5. The bottom spectra on both figures are the spectra for mass reference samples (P321, P621, or tetracosenoic acid) under the same conditions with the single HeLa cell sample analysis as the blank spectra. All mass peaks on the spectra obtained from the HeLa cell were grayed out if the corresponding masses (±5 mDa width) are also shown in the reference spectrum at the bottom of each figure. All the extracted ion profiles for the masses on the HeLa cell spectra were then evaluated.

### 3.3. Methanol extracted HeLa cell sample analysis

**Figure 5:**
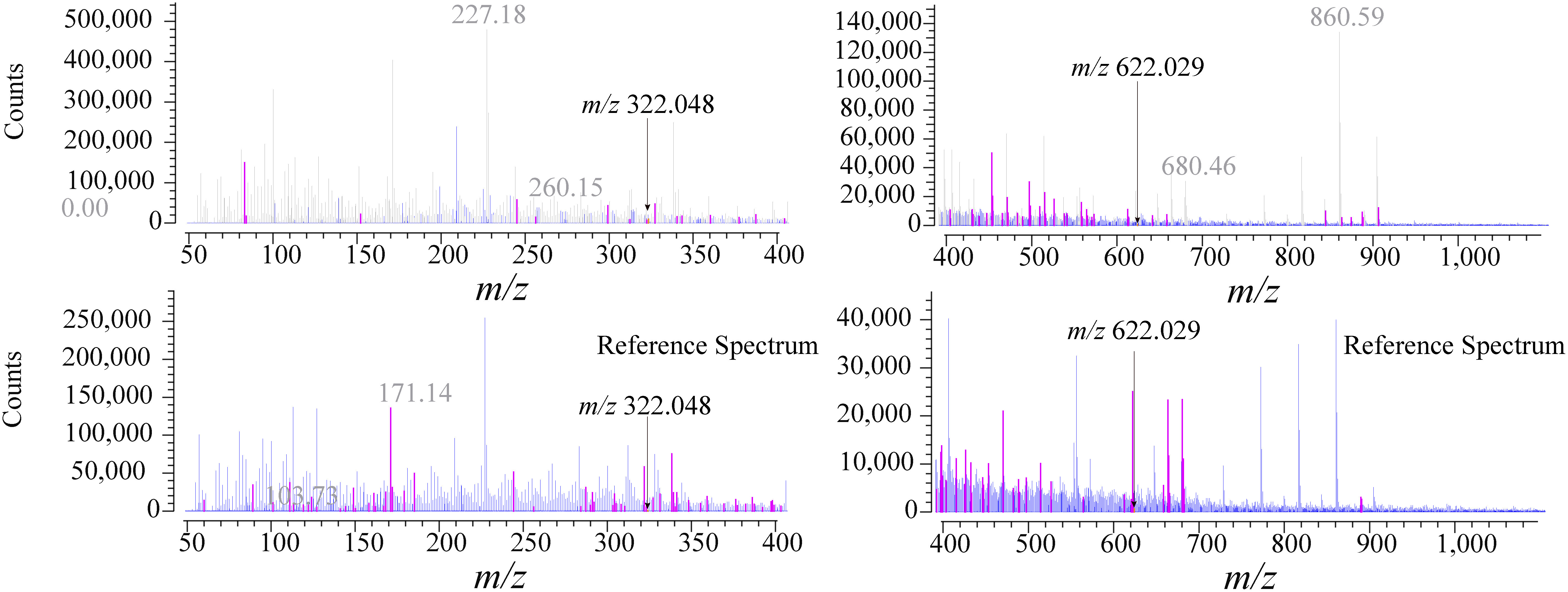
Fig. 5. Positive ion mode SFE-PTR mass spectrum of 0.2 μL (20 cells equivalent) of the methanol-extract of a cell (top). SFE-PTR mass spectrum of lock mass reference sample (bottom). The spectrum data file is available in J-STAGE Data.https://doi.org/10.50893/data.massspectrometry.21706646

**Figure 6:**
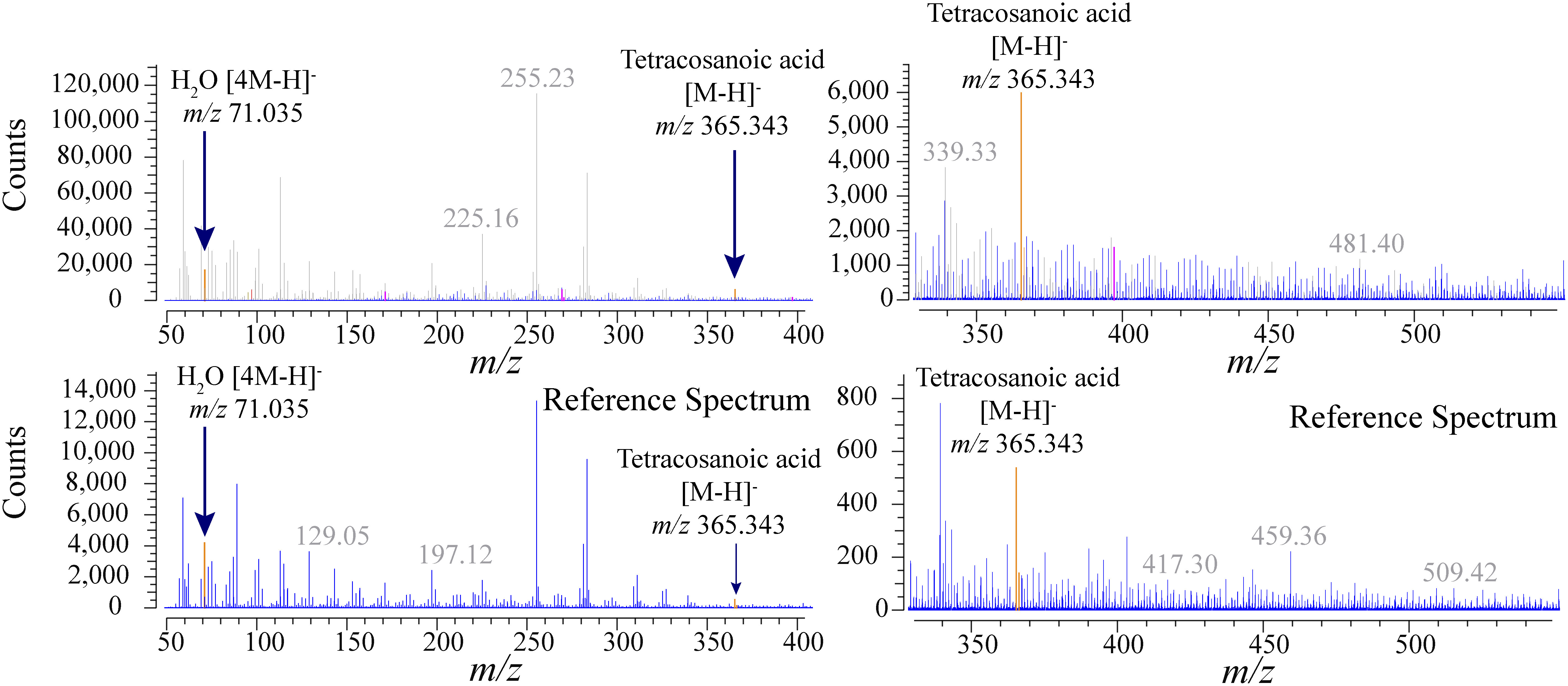
Fig. 6. Negative ion mode SFE-PTR mass spectrum of a methanol-extract equivalent to 20 cells (top). SFE-PTR spectrum of lock mass reference sample (bottom) The spectrum data file is available in J-STAGE Data. https://doi.org/10.50893/data.massspectrometry.21706652

**Figure 7:**
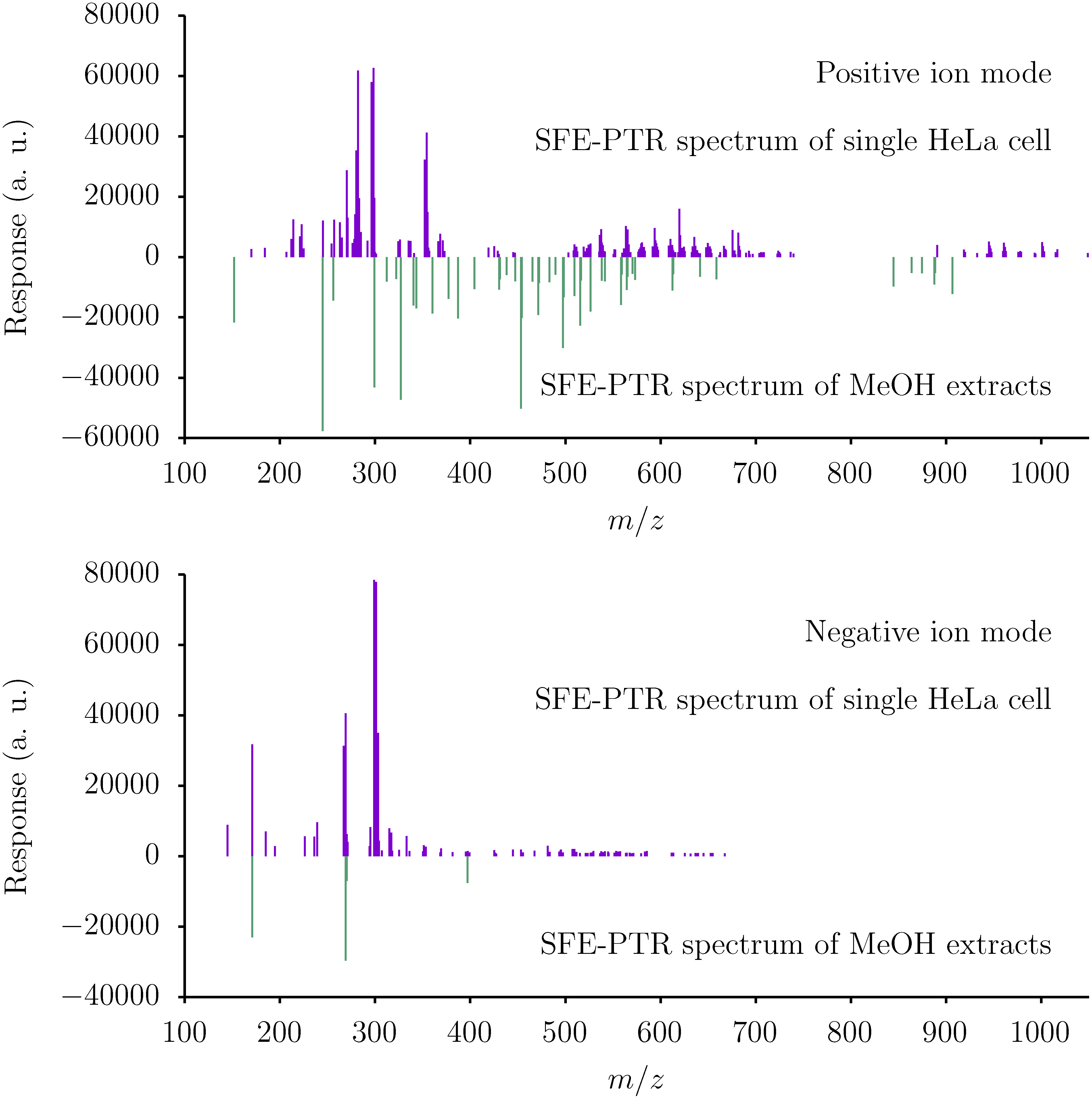
Fig. 7. Comparison of direct SFE from a single HeLa cell and a methanol-extract (20 cells equivalent). Spectra were plotted for the ions color-coded to magenta, which are assumed to be ions derived from the actual sample.

Figures 5 and 6 are the co-added SFE-PTR mass spectra for 20 HeLa cell equivalent amounts of a methanol-extracted cell (top), and their background spectra in the bottom. Color codes are the same as for previous spectra. A total of 200 (91) and 50 (4) ions were observed from the SFE of a single HeLa cell and methanol extracts in the positive (negative) ion mode. Figure 7 shows a comparison of the observed ions obtained from the SFE directory from a single HeLa cell and the methanol-extracted cell. Table 2 shows a list of matched ions that appear in both extraction methods. It is unlikely that most ions observed in SFE from a single HeLa cell were not present in the methanol-extracted cell. Recently, lipidomics researchers, M. Höring *et al.*,^22)^ reported that the extraction efficiency of lipids varies greatly depending on the polarity of the solvent being used. The present results suggest an extraction bias for lipids between intact cells and their methanol extracts. We currently do not have sufficient evidence for whether the detected ions originate from a cell or are contaminants from the environment, such as plasticizers from the vials used in the experiments.

**Table 2:** Table 2. List of ions observed in both SFE of a single HeLa cell and the methanol-extracted cell.

MeOH extracts		SFE of single cell		δ*m* (mDa)
*m*/*z*	Intensity	*m*/*z*	Intensity	
Positive ion mode				
526.422	18011	526.425	4440	−2.75
Negative ion mode				
171.137	4602.5	171.139	31818	−1.87
269.242	5924	269.245	40638	−2.82

One of the advantages of PTR ionization combined with SFE without a modifier is that it produces simple ions, such as protonated and deprotonated ions, with an occasional loss of water or hydrogen. Unlike liquid chromatography with electrospray ionization (ESI), no complex adducts, such as sodium, ammonium, acetonitrile, *etc.*, are produced. Therefore, the list of all possible ion formulae for a list of lipid structures can easily be synthesized *in silico*. A list of structure data (LMSD Database) obtained from https://www.lipidmaps.org/ was used after desalting and substituting isotope-specified atoms by using KNIME 4.6.1 with RDKit Node. The normalized structure data was saved as an SDF file, which contains 39883 unique structures for masses 1200. A list of possible ions was generated by adding a combination of formulae +H^+^, −e, and −HO^−^ for positive ions, −H^+^, +e, and +OH^−^ for negative ions. The ions that matched within a standard deviation of 3 mDa errors (*n*=3) are listed in Tables 3 and 4. Eleven ions in the positive ion mode could be assigned to chemical formulae as listed in Table 3. Most of the assigned ions do not match uniquely to the structure, even limited to LipidMaps listed structures. The ion assigned to *m*/*z* 282.279 ([C_18_H_35_NO+H]^+^) is possibly a protonated oleamide, which is a common background derived from laboratory ware^23)^; however, the corresponding SFE profile was not found in the reference spectrum and, therefore, it remains listed. A total of 10 ions in the negative ion mode were assigned to a formula as listed in Table 4. One of the ions that appeared at *m*/*z* 303.233 matches FFA C20:4, the protonated form of C_20_H_32_O_2_, off by −0.16 mDa. Its formula matches 41 molecular structures (isomers) listed in the database, thus, whether it is a single molecule or multiple co-eluting molecules is unknown. The number of counts for each sample run in triplicate experiments was 22589, 1352, and 2568 for *m*/*z* 303.233 ion, which roughly corresponds to 3.8 pmol, 230 fmol, and 440 fmol of arachidonic acid. The repeatability of the present study was around 10 to 76% RSD for the other ions but the ions for *m*/*z* 299.202, 301.217, and 303.233 are specifically large (123–135%). Although it is too early to evaluate the quantitative results in this preliminary study, it still holds the potential to permit the cell-to-cell variance to be determined, which cannot be discovered by the analysis of a whole cell homogenate. Even though the SFE is capable of selective extraction from the sample matrix, many compounds are simultaneously co-eluted and enter the analyzer, which may suppress ionization. Adding supercritical fluid chromatography (SFC) following the SFE may improve detection sensitivity and the accuracy of identification of molecules. Several configurations of online SFE coupled with SFC have been reported in the past four decades and have been shown to have a tremendous advantage in terms of enhancing analytical sensitivity and accuracy due to the fact that there is less chance of producing contaminants, rapid sample preparation, and the fact that no sample handling before SFE is needed. To establish an SFC-PTR MS interface, a study of peak dispersion in the PTR flow tube is mandatory.^17,24)^

**Table 3:** Table 3. Assigned peaks for positive ion mode obtained from the SFE of a single HeLa cell. A number followed by a ‘+’ sign represents the additional number of structures that match the formula listed in the LipidMaps database.

*m*/*z* (error (σ mDa))	Abundance	RSD	Formula		+isomers	Exact *m*/*z*
221.102 (±1.2)	2135	19%	C_9_H_16_O_6_	[M+H]^+^		221.102
263.236 (±2.7)	39475	41%	C_18_H_30_O	[M+H]^+^	+1	263.237
282.279 (±0.4)	248297	14%	C_18_H_35_NO	[M+H]^+^	+1	282.279
296.258 (±1.1)	169561	91%	C_18_H_33_NO_2_	[M+H]^+^	+1	296.258
297.084 (±3.0)	1418	33%	C_15_H_21_BrO	[M+H]^+^	+1	297.085
298.273 (±2.6)	130455	99%	C_18_H_35_NO_2_	[M+H]^+^	+1	298.274
300.063 (±2.7)	407	1%	C_16_H_12_O_6_	[M]^+^	+63	300.063
593.575 (±2.1)	31613	39%	C_38_H_75_NO_3_	[M]^+^	+2	593.574
613.176 (±2.9)	621	13%	C_27_H_32_O_16_	[M+H]^+^	+3	613.176
890.612 (±1.8)	709	10%	C_47_H_88_NO_12_P	[M+H]^+^	+1	890.612
1000.766 (±0.4)	22547	73%	C_56_H_105_NO_13_	[M+H]^+^	+2	1000.766
P321	4255	14%	C_6_H_18_N_3_O_6_P_3_	[M+H]^+^	—	322.048
P621	2868	38%	C_12_H_18_F_12_N_3_O_6_P_3_	[M+H]^+^	—	622.029

**Table 4:** Table 4. Assigned peaks for negative ion mode obtained from the SFE of a single HeLa cell. A number followed by a ‘+’ sign represents the additional number of structures that match the formula listed in the LipidMaps database.

*m*/*z* (error (σ mDa))	Abundance	RSD	Formula		+isomers	Exact *m*/*z*
171.139 (±0.3)	52787	51%	C_10_H_20_O_2_	[M−H]^−^	+1	171.139
194.994 (±2.0)	1561	76%	C_9_H_8_OS_2_	[M−H]^−^	+1	194.994
239.201 (±1.4)	6906	52%	C_15_H_28_O_2_	[M−H]^−^	+1	239.202
294.183 (±1.5)	3139	15%	C_17_H_26_O_4_	[M]^−^		294.184
299.201 (±2.2)	20520	123%	C_20_H_28_O_2_	[M−H]^−^	+1	299.202
301.217 (±0.7)	19337	135%	C_20_H_30_O_2_	[M−H]^−^	+1	301.217
303.233 (±0.5)	8836	135%	C_20_H_32_O_2_	[M−H]^−^	+42	303.233
381.374 (±1.6)	279	9%	C_25_H_50_O_2_	[M−H]^−^	+8	381.374
425.340 (±1.1)	1659	20%	C_27_H_43_N_3_O	[M]^−^		425.341
455.390 (±1.6)	1110	54%	C_31_H_52_O_2_	[M−H]^−^	+1	455.389
Tetracosenoic acid	10299	40%	C_24_H_46_O_2_	[M−H]^−^	—	455.389

## 4. CONCLUSIONS

We report on an evaluation of SFE-PTR MS for analyzing small lipophilic molecules contained in a single HeLa cell. About 200/91 ions were the observed in positive/negative ion mode mass spectra. In contrast, fewer ions were observed from methanol extracts of HeLa cells, and most of the ions observed in both SFE and methanol extracts did not match, although we have not yet addressed those differences. The ions of 11/10 (positive/negative) match the chemical formula obtained from the lipids database, which contains 39,883 unique structures. Each of the assigned ions has a range of about 1,000 to 60,000 ion counts, which demonstrates that the method described here has great potential for detecting small molecules in a single cell. Adding SFC separation following the SFE would be expected to improve molecular identification performance since SFC has a high column efficiency. Although, more improvement in the instrumentation for an SFC-PTR ion source is needed to allow for a narrow peak to be visualized in the SFC chromatogram, which is typically less than 0.5 s.

Furthermore, the process used in this study does not fully resolve the issue of false positive results by unidentified contaminants derived from laboratory ware used in the sample processing. The present method shows, however, the potential of the method for use in the analysis of metabolites from a single cell.

## Data Availability

The spectrum data files of Figs. 3–6 are available in J-STAGE Data.

## Notes

Mass Spectrom (Tokyo) 2022; 11(1): A0112
